# Effects of Body Mass Index on Task-Related Oxygen Uptake and Dyspnea during Activities of Daily Life in COPD

**DOI:** 10.1371/journal.pone.0041078

**Published:** 2012-07-17

**Authors:** Anouk W. Vaes, Frits M. E. Franssen, Kenneth Meijer, Martijn W. J. Cuijpers, Emiel F. M. Wouters, Erica P. A. Rutten, Martijn A. Spruit

**Affiliations:** 1 Program Development Centre, CIRO +, Horn, the Netherlands; 2 Physiotherapy, CIRO +, Horn, the Netherlands; 3 Department of Human Movement Science, School for Nutrition, Toxicology and Metabolism of MUMC +, Maastricht, the Netherlands; 4 Respiratory Medicine, MUMC +, Maastricht, the Netherlands; Clinica Universidad de Navarra, Spain

## Abstract

**Background:**

Patients with COPD use a higher proportion of their peak aerobic capacity during the performance of domestic activities of daily life (ADLs) compared to healthy peers, accompanied by a higher degree of task-related symptoms. To date, the influence of body mass index (BMI) on the task-related metabolic demands remains unknown in patients with COPD. Therefore, the aim of our study was to determine the effects of BMI on metabolic load during the performance of 5 consecutive domestic ADLs in patients with COPD.

**Methodology:**

Ninety-four COPD patients and 20 healhty peers performed 5 consecutive, self-paced domestic ADLs putting on socks, shoes and vest; folding 8 towels; putting away groceries; washing up 4 dishes, cups and saucers; and sweeping the floor for 4 min. Task-related oxygen uptake and ventilation were assessed using a mobile oxycon, while Borg scores were used to assess task-related dyspnea and fatigue.

**Principal Findings:**

1. Relative task-related oxygen uptake after the performance of domestic ADLs was increased in patients with COPD compared to healthy elderly, whereas absolute oxygen uptake is similar between groups; 2. Relative oxygen uptake and oxygen uptake per kilogram fat-free mass were comparable between BMI groups; and 3. Borg symptom scores for dyspnea en fatigue were comparable between BMI groups.

**Conclusion:**

Patients with COPD in different BMI groups perform self-paced domestic ADLs at the same relative metabolic load, accompanied by comparable Borg symptom scores for dyspnea and fatigue.

## Introduction

Chronic obstructive pulmonary disease (COPD) is not only characterised by pathological changes of the respiratory system, but has also significant systemic consequences like involuntary weight loss and skeletal muscle dysfunction [1], [2]. These systemic changes adversely affect patients exercise performance, quality of life and prognosis [2].

Involuntary weight loss and muscle wasting have traditionally been an active field of interest in patients with COPD, but overweight and obesity are increasingly common [1], [3]. An overweight or obese body mass index (BMI, kg/m^2^) is strongly associated with a decreased compliance of the respiratory system, a restrictive lung function, increased airway resistance, and increased work of breathing [1]. This can result in a worsening of dyspnea, increased physical limitations and decreased quality of life [1]. On the other hand, being overweight or obese has been associated with less osteoporosis [4] and a decreased mortality among patients with severe COPD; a phenomenon commonly referred to as the ‘obesity paradox’ [1], [2].

Functional exercise performance and physical activity levels have been shown to be reduced in patients with COPD compared to healthy subjects, even in the earliest stage of the disease [3], [5]–[9]. Moreover, overweight and obese COPD patients have a significantly shorter six minute walk distance compared to normal and low BMI patients [10], [11]. However, walk-work (the product of six minute walk distance in meters and body weight in kilogram) is similar between obese en non-obese COPD patients [10]. This suggests that the reduced functional exercise performance reflects, at least in part, the increased work burden of overweight and obesity. Indeed, obesity has been associated with reduced physical activity levels in patients with COPD, irrespective of the degree of lung function impairment [12].

**Table 1:** Characteristics.

	Healthy subjects (n = 20)	COPD patients (n = 94)
Men (%)	60	61
Age (years)	62.1 (5.6)	60.4 (9.3)
FEV1 (L)	3.34 (0.61)	1.42 (0.57)^†^
FEV1 (% predicted)	119.4 (24.2)	51.2 (19.1)^†^
FEV1/FVC (%)	76.8 (4.6)	42.5 (13.0)^†^
TLCO (%)	–	56.7 (19.8)
*P*aO2 (kPa)^*^	–	9.75 (1.24)
*P*aCO2 (kPa)	–	5.20 (0.66)
*S*aO2 (%)	–	95.2 (2.1)
GOLD stage I/II/III/IV (n)	–	5/36/37/16
MRC grade 1/2/3/4/5 (n)	–	7/28/35/12/12
BODE score (points)	–	2.88 (1.89)
Body weight (kg)^**^	79.2 (12.3)	69.8 (17.3)^†^
Height (cm)	171.1 (7.7)	168.4 (9.7)
Body mass index (kg/m^2^)	27.0 (3.0)	24.5 (5.1)^†^
FFM (kg)	55.6 (9.9)	45.5 (9.1)^†^
FFMI (kg/m^2^)	18.9 (2.3)	16.0 (2.3)^†^
Women <15 kg/m^2^ (%)	25.0	62.2
Men <16 kg/m^2^ (%)	0.0	45.5
Charlson CMI (points)	0.0	2.0 (1.4)^†^
Charlson CMI 2 (points)	1.7 (0.7)	3.7 (2.0)^†^
≥1 co-morbidities (%)	0.0	56.1^†^
Peak VO2 (mL/min)	2155 (770)	1202 (360)^†^
Peak VO2 (mL/min/kg BW)	27.5 (10.2)	17.5 (4.4)^†^
Peak VO2 (mL/min/kg FFM)	38.8 (12.2)	26.4 (6.0)^†^
Peak VE (L)	87.3 (28.2)	47.4 (14.6)^†^
Peak VE (% MVV)	65.4 (17.1)	89.8 (26.1)^†^
Peak HR (bpm)	154 (18.9)	132 (20.0)^†^
Peak HR (% max HR)	97.5 (10.7)	83.0 (11.5)^†^
Borg dyspnoea (points)	5.5 (2.2)	6.6 (2.1)^†^
Borg fatigue (points)	5.7 (2.2)	5.9 (2.2)

Results are presented as mean (standard deviation). FEV_1_  =  forced expiratory volume in the first second; L  =  litre; FVC  =  forced vital capacity; kg  =  kilogram; kg/m^2^  =  kilogram per squared meters; Charlson CMI  =  Charlson co-morbidity index [19]; Charlson CMI 2 =  Charlson age comorbidity index [20]; 6MWD  =  six-minute walking distance; VO2 =  oxygen uptake; mL  =  millilitre; min  =  minute; BW  =  body weight; FFM  =  fat free mass; VE  =  minute ventilation; MVV  =  maximum voluntary ventilation; HR  =  heart rate; bpm  =  beats per minute. ^*^1 kPa  = 7.5 mm Hg; ^**^1 kilogram  = 2.2046 pounds; ^†^p≤0.05 vs. healthy subjects.

Patients with COPD use a higher proportion of their peak aerobic capacity during the performance of simple domestic ADLs compared to healthy peers, accompanied by a higher degree of task-related dyspnea and fatigue [7], [13]–[16]. These studies did not study the possible effects of BMI on task-related oxygen uptake and symptoms in patients with COPD. Therefore, the influence of BMI on the task-related metabolic demands remains unknown in patients with COPD. Nevertheless, earlier was already shown that oxygen uptake and the ventilatory response are dependent on the corporal size. So, it seems reasonable to hypothesize that patients with low or normal BMI have a lower task-related oxygen uptake and ventilation during domestic activities in daily life compared to overweight and obese COPD patients. This is probably accompanied by lower Borg symptom scores, due to lower metabolic and ventilatory requirements during weight-bearing activities of daily life [10], [11]. Therefore, the aim of our study was to determine the effects of BMI on metabolic load during the performance of 5 consecutive domestic activities of daily life in patients with COPD.

**Table 2:** Baseline characteristics by BMI categories.

	<21 kg/m^2^ (n = 24)	21–25 kg/m^2^ (n = 31)	25–30 kg/m^2^ (n = 26)	>30 kg/m^2^ (n = 13)
Men (%)	50	45	77^*^+	85^*^+
Age (years)	57.2 (7.8)	59.5 (9.1)	66.3 (8.7)^†^+	56.8 (8.8)^#^
FEV1 (L)	1.25 (0.61)	1.37 (0.52)	1.54 (0.64)	1.59 (0.44)
FEV1 (% predicted)	44.8 (19.9)	51.7 (18.3)	56.9 (20.1)	50.5 (15.1)
FEV1/FVC (%)	39.2 (14.7)	41.0 (11.1)	44.3 (13.4)	48.5 (12.0)
TLCO (%)	53.3 (18.6)	54.4 (19.3)	57.3 (20.4)	61.1 (19.0)
*P*aO2 (kPa)^*^	9.79 (1.41)	9.65 (1.05)	9.77 (1.17)	9.85 (1.56)
*P*aCO2 (kPa)	5.33 (0.92)	5.14 (0.56)	5.11 (0.53)	5.34 (0.62)
*S*aO2 (%)	94.7 (3.2)	95.4 (1.6)	95.4 (1.5)	95.3 (1.8)
GOLD stage I/II/III/IV (n)	1/6/6/11	1/14/13/3	2/11/11/2	1/5/6/1
MRC grade 1/2/3/4/5 (n)	3/5/9/4/3	2/10/11/5/3	1/9/10/3/3	1/4/6/1/1
BODE score (points)	4.2 (2.0)	2.4 (1.4)^†^	2.4 (2.0)^†^	2.5 (1.5)^†^
Body weight (kg)^**^	52.6 (7.9)	64.9 (7.7)^†^	76.7 (11.0)^†^+	99.7 (7.8)^†^+^#^
Height (cm)	167.0 (9.2)	167.7 (9.7)	168.5 (10.5)	172.4 (8.6)
Body mass index (kg/m^2^)	18.8 (1.7)	23.0 (1.0)^†^	26.9 (1.4)^†^+	33.7 (3.6)^†^+^#^
FFM (kg)	39.0 (5.4)	42.9 (5.7)	48.5 (8.8)^†^+	59.3 (6.7)^†^+^#^
FFMI (kg/m^2^)	14.0 (1.2)	15.2 (1.3)^†^	17.0 (1.6)^†^+	20.0 (1.5)^†^+^#^
Women <15 kg/m^2^ (%)	75.0	76.5	16.7	0.0
Men <16 kg/m^2^ (%)	91.7	71.4	20.0	0.0
Charlson CMI (points)	1.7 (0.9)	1.9 (1.1)	2.5 (1.8)	2.1 (1.9)
Charlson CMI 2 (points)	3.0 (1.5)	3.4 (1.8)	4.8 (2.2)^†^	3.4 (2.6)
≥1 co-morbidities (%)	57.6	55.6	56.7	53
6MWD (m)	487 (111)	500 (96)	483 (114)	485 (108)
Peak VO2 (mL/min)	1009 (306)	1172 (330)	1290 (343)^†^	1458 (374)^†^
Peak VO2 (mL/min/kg BW)	19.2 (4.9)	18.0 (4.3)	16.8 (3.6)	14.6 (3.7)^†^
Peak VO2 (mL/min/kg FFM)	25.7 (6.1)	27.3 (6.5)	26.7 (5.2)	24.7 (6.1)
Peak VE (L)	42.6 (11.2)	45.7 (15.6)	50.6 (15.3)	53.8 (14.1)
Peak VE (% MVV)	96.9 (31.3)	83.5 (17.5)	91.5 (29.0)	88.0 (25.4)
Peak HR (bpm)	130 (17.8)	137 (19.8)	130 (19.4)	131 (25.5)
Peak HR (% max HR)	80.1 (10.8)	85.2 (10.3)	84.7 (12.5)	79.8 (12.6)
Borg dyspnoea (points)	6.5 (2.5)	7.2 (2.0)	6.2 (1.7)	6.2 (2.4)
Borg fatigue (points)	5.6 (2.5)	6.4 (2.6)	5.6 (1.6)	5.6 (2.0)

Results are presented as mean (standard deviation). FEV_1_  =  forced expiratory volume in the first second; L  =  litre; FVC  =  forced vital capacity; kg  =  kilogram; kg/m^2^  =  kilogram per squared meters; Charlson CMI  =  Charlson co-morbidity index [19]; Charlson CMI 2 =  Charlson age comorbidity index [20]; 6MWD  =  six-minute walking distance; VO2 =  oxygen uptake; mL  =  millilitre; min  =  minute; BW  =  body weight; FFM  =  fat free mass; VE  =  minute ventilation; MVV  =  maximum voluntary ventilation; HR  =  heart rate; bpm  =  beats per minute. ^*^1 kPa  = 7.5 mm Hg; ^**^1 kilogram  = 2.2046 pounds; ^†^p<0.05 vs. <21 kg/m^2^ +p<0.05 vs. 21–25 kg/m^2^^#^p<0.05 vs. 25–30 kg/m^2^.

## Methods

One hundred COPD patients and 25 healthy peers volunteered to participate in the present study, which was approved by the institutional review board of the Maastricht University Medical Centre (MEC08-3-032). Patients were recruited at CIRO + in Horn (the Netherlands) before the start of a comprehensive pulmonary rehabilitation program [17] and had not participated in clinical studies before [7]. Healthy volunteers were recruited through posters and among healthy subjects who had participated in previous trials. At enrolment, written informed consent was obtained from potential participants and study eligibility was determined.

**Figure 1:**
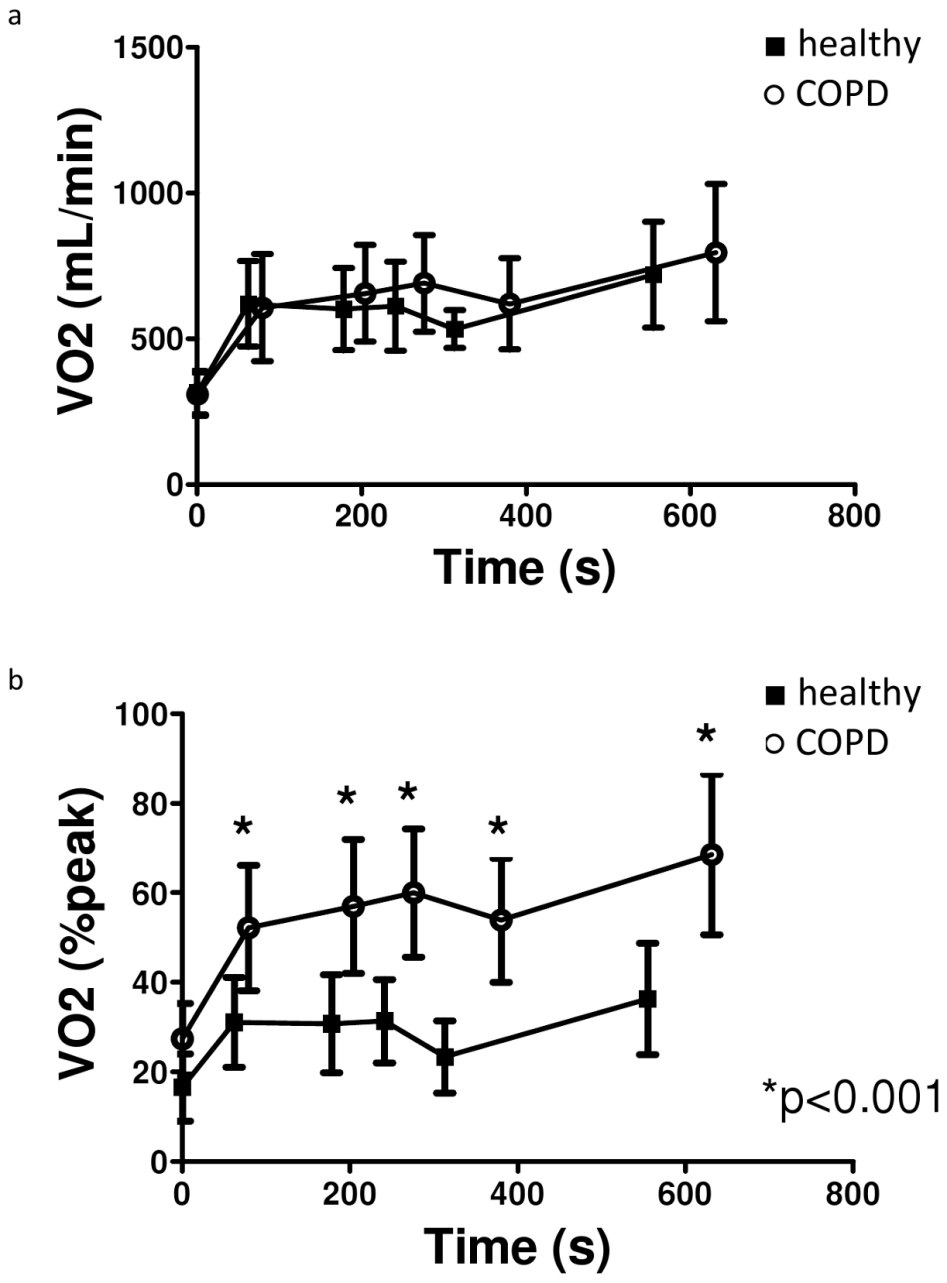
Task-related oxygen uptake in patients with COPD and healthy controls. a. Absolute task-related oxygen uptake (mL/min). b. Relative task-related oxygen uptake (%peakVO_2_).

Patients were clinically stable (no exacerbation in past 4 weeks) and were on various (non-) pulmonary drug therapies (Table S1). None of the healthy subjects used physician-prescribed drugs. Exclusion criteria were the use of long-term oxygen therapy, neuromuscular co-morbidities, a cardiac pacemaker and/or an implantable cardioverter defibrillator and the disability to perform the domestic ADLs were included in the study. All tests were in accordance with the World Medical Association declaration of Helsinki.

**Figure 2:**
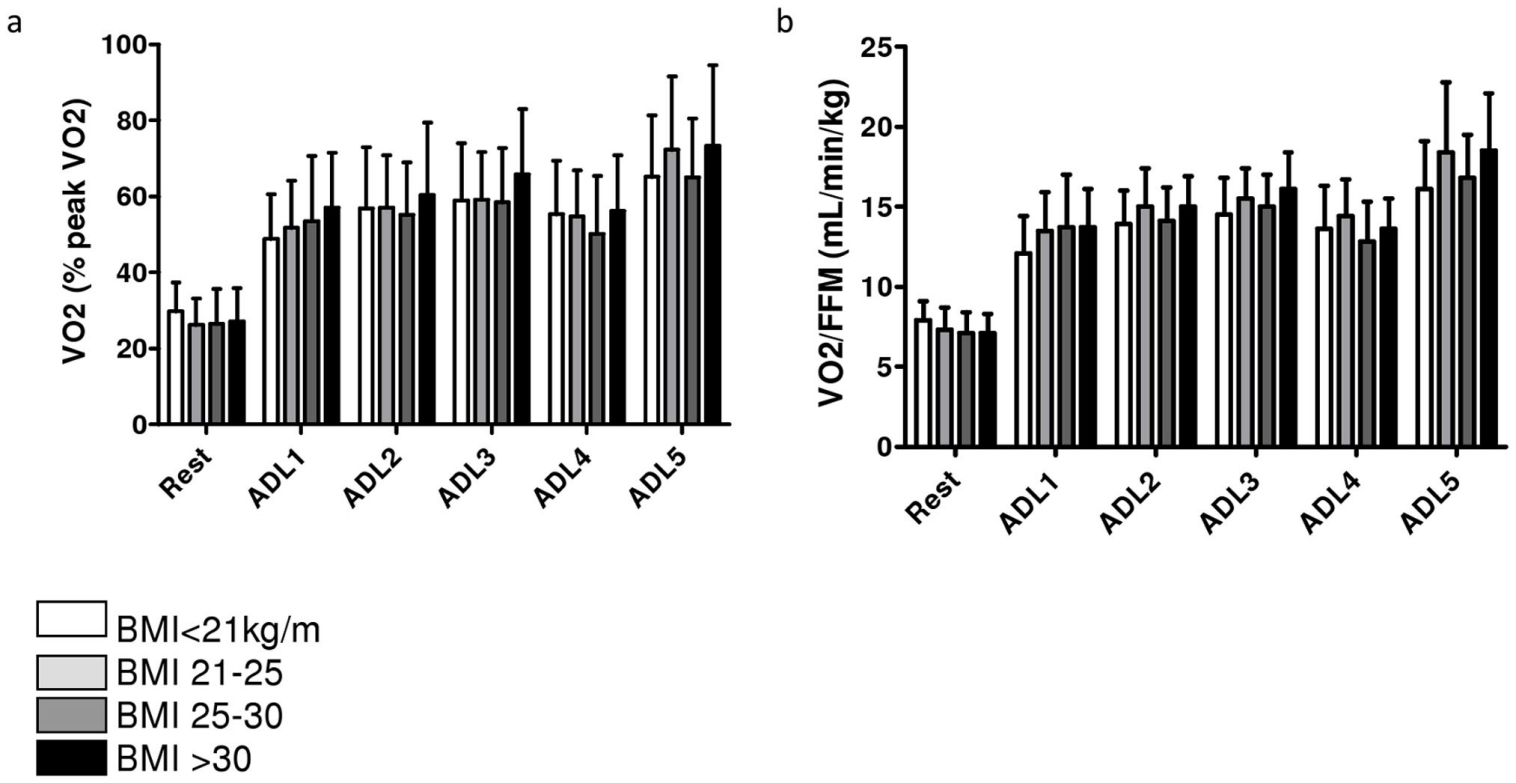
Task-related oxygen uptake in patients with COPD. a. Relative task-related oxygen uptake (%peakVO_2_). b. Task-related oxygen uptake per kilogram FFM (mL/min/kg FFM).

### Clinical Phenotyping

General demographics, co-morbidities, post-bronchodilator pulmonary function, dyspnea, body composition and peak aerobic capacity were assessed as described previously [14], [18]. Co-existing morbidities were scored using the Charlson Comorbidity Index and the Charlson Age Comorbidity Index, as described before [7], [19], [20]. The scores were based on patients’ self-reported co-morbidities and data from medical records.

**Figure 3:**
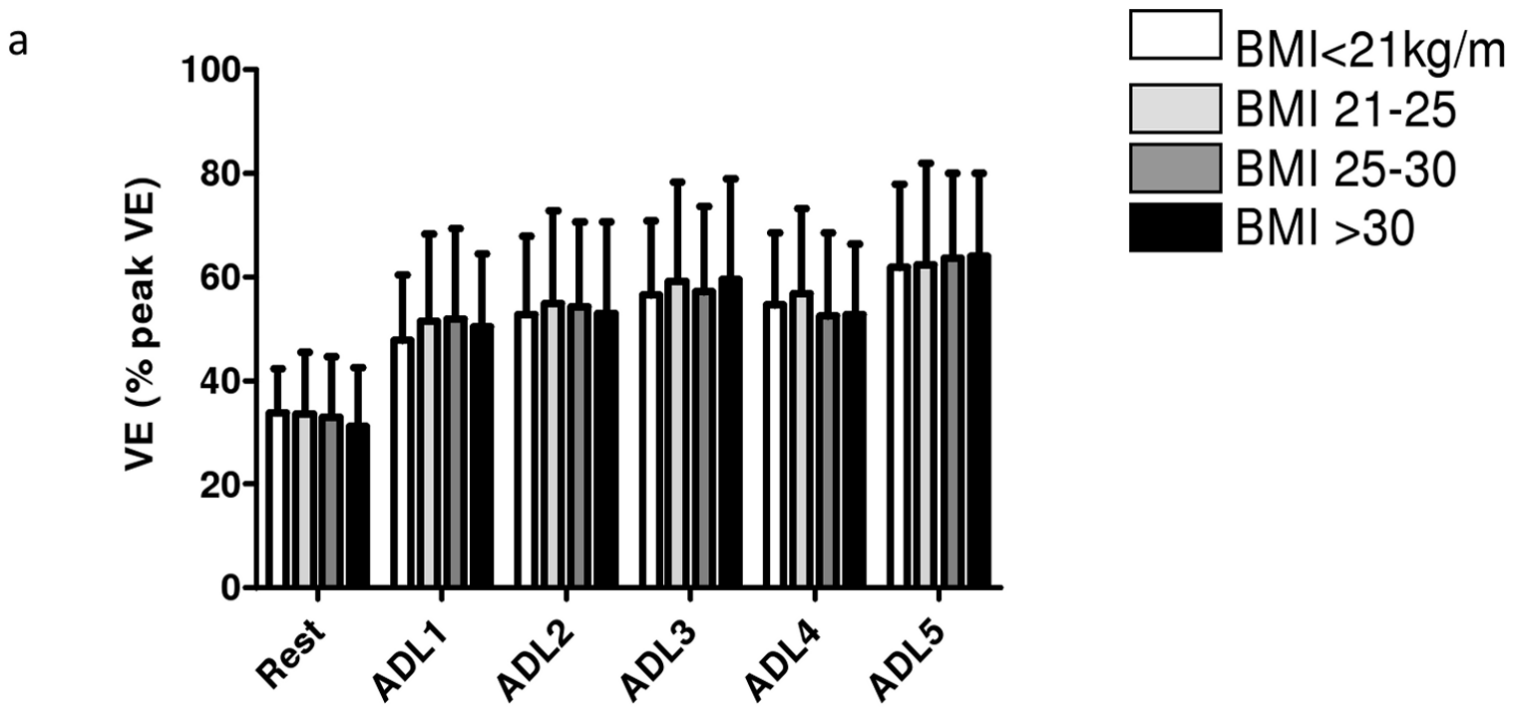
Task-related ventilation in patients with COPD. a. Relative task-related ventilation (%peakVE).

### Domestic ADLs

Participants performed 5 domestic ADLs in the kitchen of the Dept. of Occupational Therapy at CIRO + as described before [7]: putting on 2 socks (sitting in chair), 2 shoes (sitting in chair) and a vest (standing, ADL1); folding 8 towels (standing, ADL2); putting away groceries (*e.g.*, 6 cans of beans of 400 grams each) in a cupboard (standing and walking, ADL3); washing up 4 dishes, 4 cups and 4 saucers (standing, ADL4); and sweeping the floor for 4 minutes (standing and walking, ADL5). These ADLs have all be identified as problematic valued life activities in patients with COPD [9], [21]. Participants were asked to perform ADLs at their own pace; and all ADLs were performed consecutively and, unlike to our previous study [7], without four minute rest intervals between ADLs.

**Figure 4:**
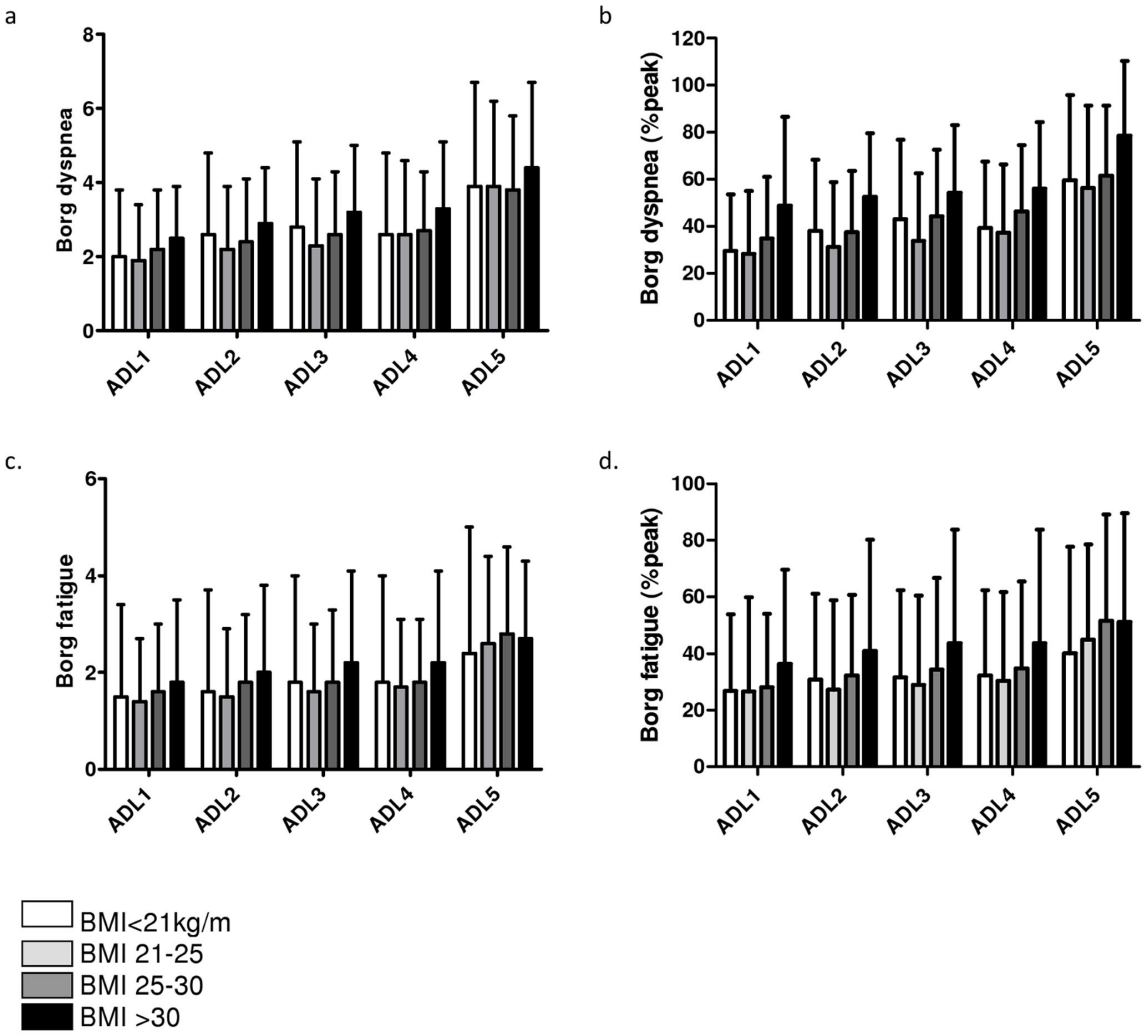
Borg symptom scores after performance of domestic ADLs. a. absolute Borg dyspnea scores. b. relative Borg dyspnea scores (%peak). c. absolute Borg fatigue scores. d. relative Borg fatigue scores (%peak).

Online breath-by-breath calculations of oxygen uptake and ventilation were recorded using a mobile oxycon (Oxycon Mobile, CareFusion, San Diego, CA, USA) which provides reliable measurements of oxygen uptake and ventilation. A mobile oxycon has been used before in patients with moderate to very severe COPD and in patients with chronic heart failure without adverse events [7], [15], [22]–[24].

Heart rate was monitored using a Polar belt. Moreover, all participants were asked to score the degree of dyspnea and fatigue at the beginning ADL1 and at the end of all ADLs using a modified Borg symptom score ranging from 0 (no symptoms) to 10 points (worst symptoms). For unknown reasons a download of the data failed in 6 patients and 5 healthy subjects. Therefore, the final group comprised 94 COPD patients and 20 healthy subjects.

*A priori,* relative task-related oxygen uptake during ADLs (ie, task-related oxygen uptake expressed as a proportion of the pre-determined peak aerobic capacity) was chosen as the primary outcome. Task-related oxygen uptake (mL/min) per kilogram fat-free mass (FFM, using whole-body dual-energy x-ray absorptiometry (DEXA, GE Medical Systems Lunar Prodigy, Madison, USA)), task-related ventilation (expressed as a proportion of pre-determined peak ventilation), time to accomplish the first four ADLs (ADL5 was always set at 4 min), and Borg symptom scores for dyspnea and fatigue at the end of each ADL were chosen as secondary outcomes.

### Statistics

Data are presented as mean and standard deviation, unless noted otherwise. Student’s t-test was used to determine differences between patients with COPD and healthy subjects. Patients were divided according to BMI: low BMI: <21 kg/m^2^; normal BMI: 21 kg/m^2^ to 25 kg/m^2^; overweight BMI: >25 to 30 kg/m^2^; or obese BMI: >30 kg/m^2^. Differences between BMI groups were compared using analysis of variance (ANOVA) and Bonferroni post hoc comparisons. Chi squared (with Cramer’s V measure for strength of association) was used to assess the differences in gender distribution between groups. *A priori,* the level of significance was set at ≤0.05. Data were analyzed with SPSS, version 19.0.

## Results

### Characteristics

Subject characteristics are listed in table 1. On average, patients had mild to very severe COPD, a normal BMI and a poor exercise capacity. Patients had a significantly lower BMI and FFM compared to healthy subjects. Moreover, patients had higher scores on the (age-adjusted) Charlson Comorbidity Index. Most common co-morbidities were cardiovascular diseases. Furthermore, 19.6% of the COPD patients had a history of orthopaedic problems. Present comorbidities had no significant effect on exercise tolerance.

Patients with COPD had a significantly lower peak aerobic capacity (mL/min), peak heart rate (beats/min), and peak ventilation (L) than healthy subjects. Peak Borg dyspnea scores of patients were significantly higher than those of healthy subjects, whereas peak Borg fatigue scores were comparable between groups (Table 1).

Characteristics of the patients with COPD per BMI group are listed in table 2. Results showed no significant differences in lung function between BMI groups; while, obviously, there were significant differences in body composition. Peak aerobic capacity (mL/min) was significantly lower in low BMI patients compared to those with an overweight and obese BMI. Peak ventilation, peak heart rate, and peak Borg symptom scores were comparable between BMI groups (Table 2).

### Domestic ADLs

#### Patients with COPD *versus* healthy subjects

Basal values before performance of ADL-protocol as well as absolute task-related oxygen uptake (mL/min) at the end of the 5 ADLs were similar between COPD patients and healthy peers (Figure 1a). Consequently, patients performed the domestic ADLs at a significantly higher proportion of their peak aerobic capacity compared to healthy subjects: mean difference (95%CI) ADL1∶21.1 (14.6–27.6)%; ADL2∶26.2 (19.2–33.2)%; ADL3∶28.5 (21.8–35.3)%; ADL4∶30.4 (24.1–36.8)% and ADL5∶32.4 (24.0–40.7)%; all p<0.001 (Figure 1b). *See* Text S1.

#### COPD patients stratified by body mass index

All patients were able to complete ADL1 to ADL4 without stops, with no significant differences in time to complete the domestic tasks between BMI groups (figure S2). Thirty patients with COPD needed 20 to 145 s rest before they were able to start ADL5, while seven patients were not able to complete ADL5 due to exertional dyspnea. No differences were found between BMI groups. Moreover, there are no significant difference in task-related metabolic load between patients who did rest before start of ADL 5 and patients who did not.

Task-related oxygen uptake expressed as a percentage of peak aerobic capacity was comparable between BMI groups at rest and after performance of all ADLs (Figure 2a). Moreover, oxygen uptake per kilogram FFM was also comparable between BMI groups for all ADLs (Figure 2b).

Relative task-related ventilation (ventilation expressed as a proportion of the pre-determined peak ventilation) was comparable between BMI groups at rest and after performance for all ADLs (Figure 3a).

Although Borg scores for dyspnea appeared lower in low BMI patients compared to obese COPD patients, these differences were non-significant (Figure 4a). Moreover, relative Borg dyspnea scores (expressed as percentage of peak Borg scores during CPET) were comparable between BMI groups (Figure 4b). Borg scores for fatigue (absolute en relative) were also comparable between BMI groups (Figure 4c and 4d).

## Discussion

Our results demonstrate differences in relative task-related oxygen uptake after the performance of five consecutive domestic ADLs between patients with COPD and healthy peers, whereas absolute oxygen uptake is similar between groups. Moreover, the present study is the first to demonstrate that relative oxygen uptake, oxygen uptake per kilogram FFM and relative ventilation during the performance of domestic ADLs were comparable between COPD patients with a low, normal, overweight or obese BMI. Moreover, Borg symptom scores for dyspnea en fatigue were comparable between BMI groups.

Previously was already reported that relative task-related oxygen uptake after the performance of domestic ADLs was increased in patients with COPD compared to healthy elderly [7], [13], [15], [16]. Our results show similar results between patients with COPD and healthy subjects during five consecutive ADLs. This may explain why patients with COPD experience more limitations in their daily life [9].

An increased BMI is associated with an increased work of breathing, increased breathing resistive load and worse exertional dyspnea [1]. On the other hand, obesity is associated with decreased resting and dynamic lung volumes [25], [26], which would positively affect exercise performance and perceived dyspnea. Indeed, overweight and obese patients with COPD did not experience greater dyspnea and exercise limitation during cycle ergometry compared to normal weight patients with comparable degree of airflow limitation [10], [27]. We also found no significant differences in dyspnea after performance of domestic ADLs between obese and non-obese COPD patients (Figure 4).

Previous studies showed that underweight COPD patients, particularly those with an abnormal low FFM, have an impaired peripheral muscle function and a poor exercise capacity [28]. Therefore, underweight patients may experience more limitations during the performance of domestic ADLs compared to normal weight COPD patients. In the presents study, underweight COPD patients had a significantly lower absolute oxygen uptake and ventilation during the performance of domestic ADLs compared to overweight or obese patients. This may indicate that obese and overweight patients experience more functional impairments in their daily life, probably caused by increases in the energetic cost of moving heavier limbs during activities and increased work of breathing [1], [25], [29], [30]. However, differences in task-related oxygen uptake and ventilation between BMI groups disappeared after correction for body weight or fat-free mass. These findings are corroborated by an *a posteriori* analysis of a previous study in which task-related oxygen uptake was assessed during five separate ADLs in 97 patients with COPD [7] (*see* Text S1 for details).

Patients with COPD use a higher proportion of their peak aerobic capacity and peak ventilation to perform domestic ADLs as compared to healthy subjects, accompanied by higher task-related Borg dyspnea scores [7], [13], [15], [16]. Moreover, task-related dynamic hyperinflation will contribute to a worsening of the task-related dyspnea in patients with COPD [22], Because of the reduced static and dynamic hyperinflation in overweight and obese COPD patients it was conceivable that these patients would experience less dyspnea during the performance of ADL compared to normal and underweight COPD patients. However, in the present study non-significant differences in task-related dyspnea were found after stratification for BMI (figure 4). This indicates that the burden of obesity restricts patients with COPD during the performance of ADL and offsets the potential beneficial influence of lower resting lung volumes.

Several methodological limitations need to be addressed. Due to the portable metabolic system we were not able to measure patients with long-term oxygen therapy, who may even experience more problematic ADLs compared to those without long-term oxygen therapy [31]. Moreover, we did not measure dynamic hyperinflation. Nevertheless, dynamic hyperinflation generally occurs during the performance of ADLs in patients with COPD [22], [32].

Fifty-six percent of the COPD patients reported ≥1 co-morbidities, which may have influenced the performance of ADLs. However, proportion of patients with co-morbidities was comparable between BMI groups and we did not found differences in task-related oxygen uptake, ventilation and dyspnea between COPD patients with and without co-morbidities (*see* Text S1 for details). This strengthens the validity of the current findings.

Stratification for BMI resulted in an unequal distribution over BMI groups. Indeed, the number of obese COPD patients seems rather low. However, the percentage of obese COPD patients in our study is comparable with the prevalence of obesity in a large primary care population of patients with COPD [33]. Moreover, the BMI groups were not comparable at baseline. However, earlier was already shown that there are no gender- or age-related differences in the metabolic demands during the performance of domestic ADLs (Vaes AW, Wouters EF, Franssen FM, Uszko-Lencer NH, Stakenborg KH, Westra M, et al. Task-Related Oxygen Uptake During Domestic Activities of Daily Life in Patients With COPD and Healthy Elderly Subjects. Chest. 2011;140(4)). Furthermore, there are no significant differences in age between patients with an obese, normal and low BMI. Therefore, the highest absolute task-related oxygen uptake and ventilation in the obese COPD group can not be explained by age-related differences.

In our study we focused on BMI, but we did not have information on fat distribution. It would be interesting to investigate whether central versus peripheral obesity exert similarly influence on the physiological response during performance of domestic ADLs.

Finally, the current findings need to be interpreted in the light of the number of comparisons that were made in the present study [34]. Nonetheless, multiple findings in the same direction, rather than a single statistically significant result, suggest that these are not due to chance alone.

To conclude, despite the additional mechanical load of obesity, relative task-related oxygen uptake and ventilation, oxygen uptake per kilogram FFM and Borg symptom scores for dyspnea and fatigue were comparable between patients with low, normal, overweight and obese BMI. Therefore, patients in different BMI groups experience similar functional limitations in the performance of domestic ADLs.

## Supporting Information

Figure S1 **Results of patients with COPD and healthy elderly subjects after performance of 5 simple domestic activities of daily life.**
(TIF)

Figure S2 **Time to accomplish ADLs.**
(TIF)

Figure S3 **Results after performance of simple domestic activities of daily life in patients with COPD after stratification for BMI.**
(TIF)

Table S1 **Pulmonary and non-pulmonary drugs.**
(DOCX)

Text S1 **Effects of Body Mass Index on Task-Related Oxygen Uptake and Dyspnea During Activities of Daily Life in COPD.**
(DOC)
