# Supplementary figures and images for: Effects of Body Mass Index on Task-Related Oxygen Uptake and Dyspnea during Activities of Daily Life in COPD
Source: PLoS One. 2012 Jul 17;7(7):e41078. doi: 10.1371/journal.pone.0041078 (PMC3398871; doi:10.1371/journal.pone.0041078)

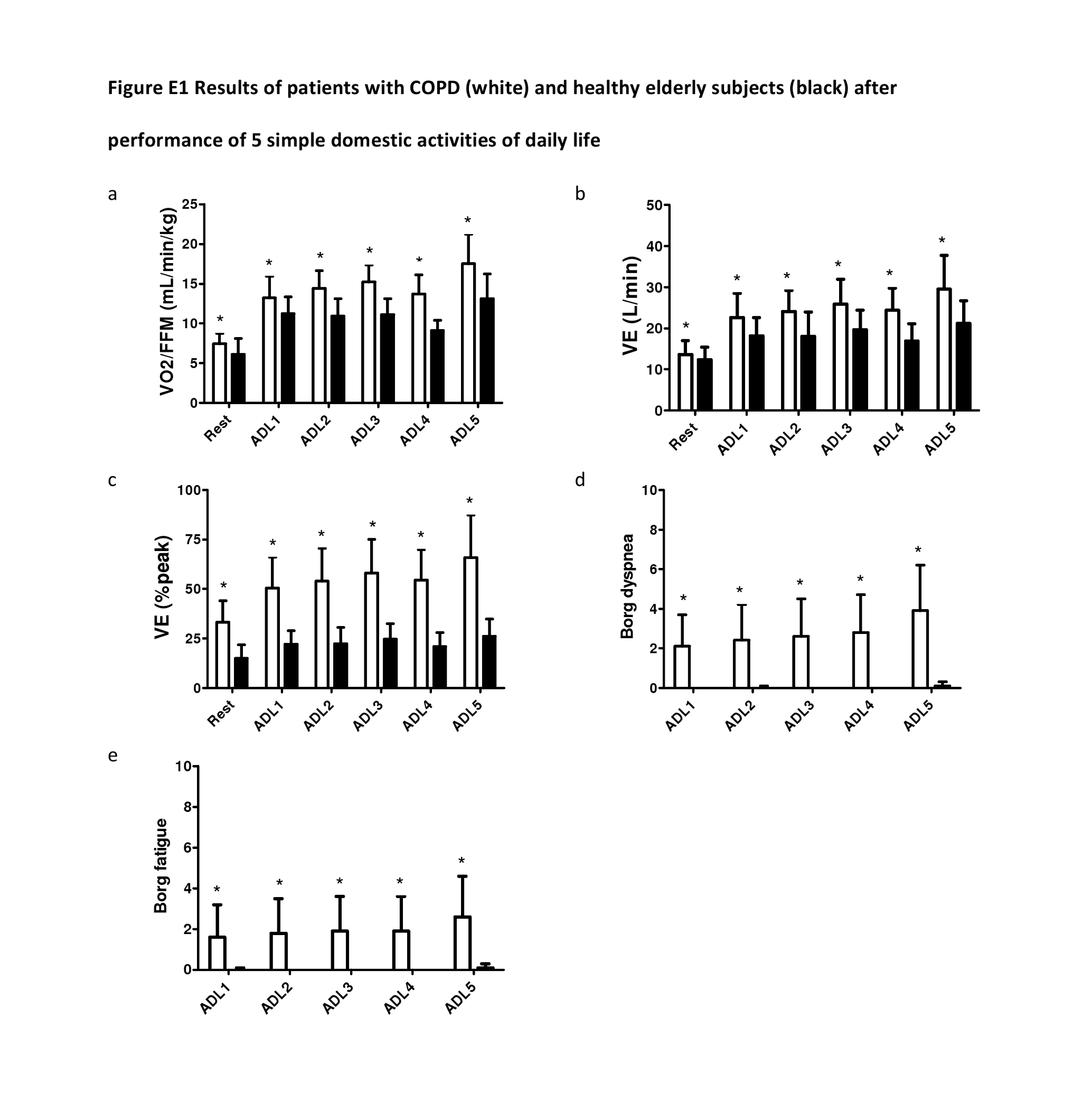

Supplement: Figure S1 — Results of patients with COPD and healthy elderly subjects after performance of 5 simple domestic activities of daily life. (TIF) [file pone.0041078.s001.tif]

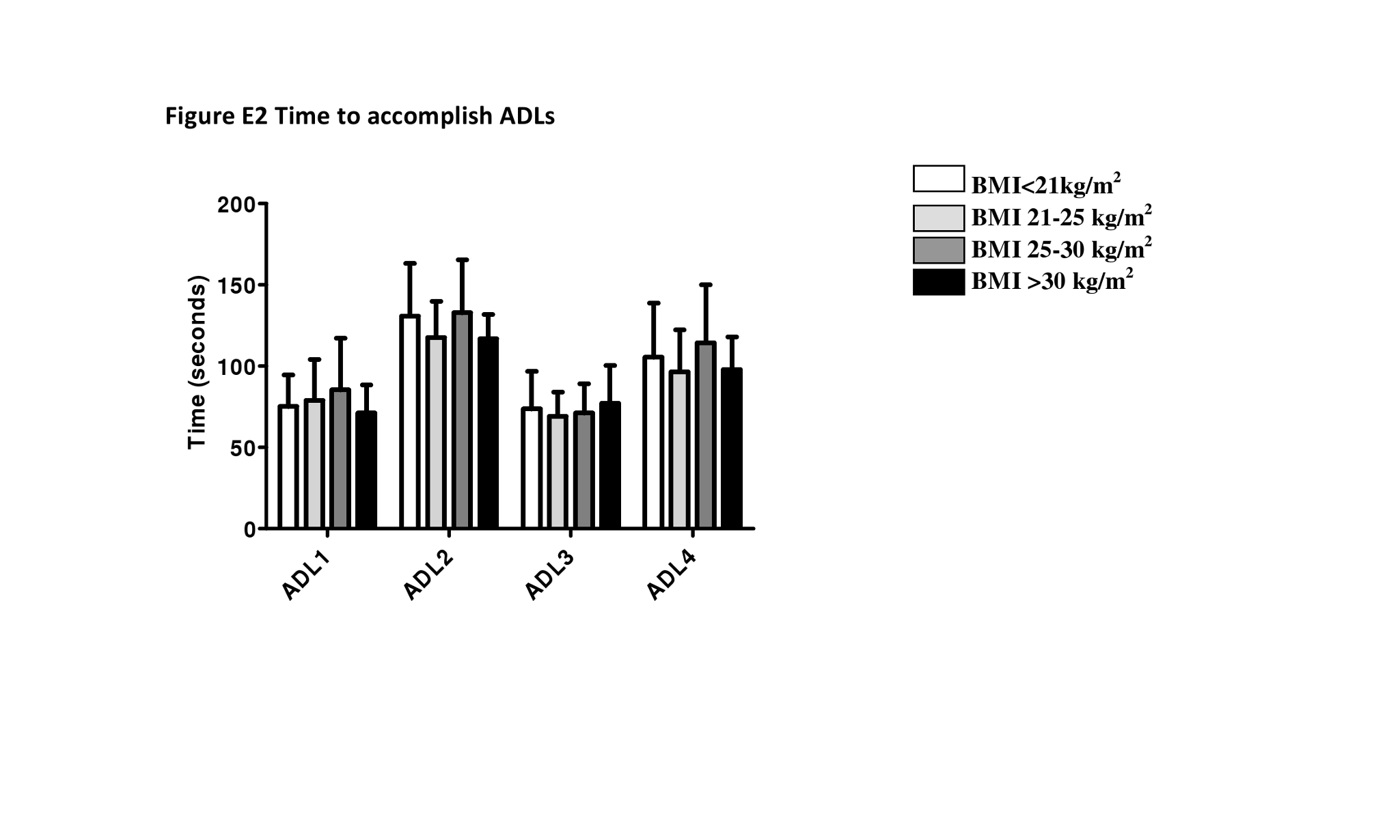

Supplement: Figure S2 — Time to accomplish ADLs. (TIF) [file pone.0041078.s002.tif]

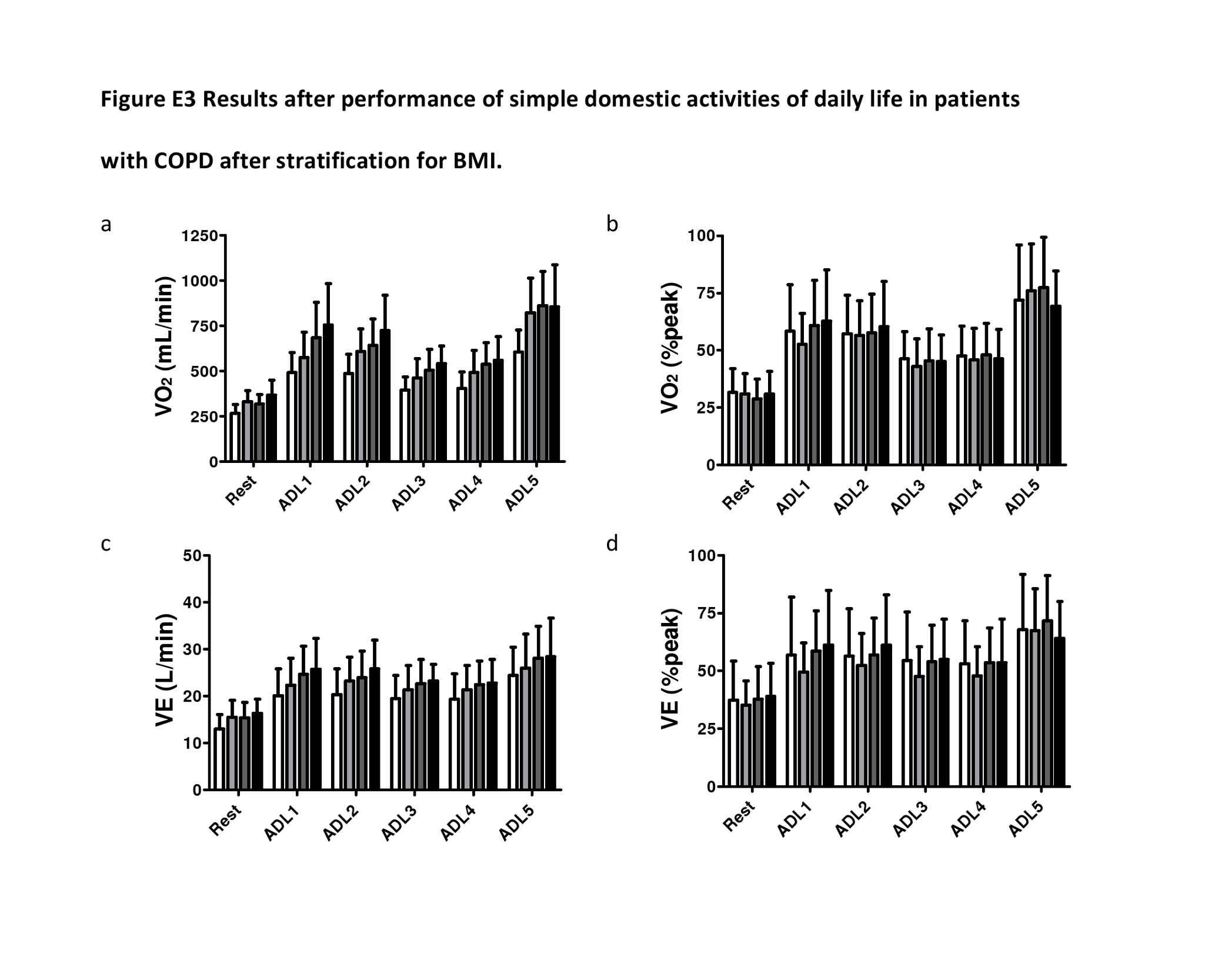

Supplement: Figure S3 — Results after performance of simple domestic activities of daily life in patients with COPD after stratification for BMI. (TIF) [file pone.0041078.s003.tif]
