# Supplementary material for: Effects of Body Mass Index on Task-Related Oxygen Uptake and Dyspnea during Activities of Daily Life in COPD
Source: PLoS One. 2012 Jul 17;7(7):e41078. doi: 10.1371/journal.pone.0041078 (PMC3398871; doi:10.1371/journal.pone.0041078)
Supplement: Table S1 — Pulmonary and non-pulmonary drugs. (DOCX) [file pone.0041078.s004.docx]

**Table S1 Pulmonary and non-pulmonary drugs**

|  | **Name of drug** | **% of 94 COPD patients** |
| --- | --- | --- |
| Pulmonary drugs | **Short-acting beta2-agonists** | *23.4* |
|  | *Short-acting anticholinergica* | *7.4* |
|  | *Short-acting combinations* | *27.7* |
|  | *Long-acting beta2-agonists* | *19.1* |
|  | *Long-acting anticholinergica* | *70.2* |
|  | *Inhaled corticosteroids* | *13.8* |
|  | *Combination ICS en LABA* | *74.5* |
|  | *Theophylline* | *16.0* |
|  | *Fluimucil* | *34.0* |
|  | *Prednison (maintenance)* | *13.8* |
|  | *Antibiotics* | *5.3* |
|  | *Singulair* | *3.2* |
| **Other drugs** | Antihypertensive agents | 27.7 |
|  | Diuretics | 17.0 |
|  | Anticoagulans | 23.4 |
|  | Cholesterol | 19.1 |
|  | Sedative/sleep medication | 23.4 |
|  | Stomach | 26.6 |
|  | Analgesic | 17.0 |
|  | Osteoporosis | 8.5 |
