# Supplementary material for: Effects of Body Mass Index on Task-Related Oxygen Uptake and Dyspnea during Activities of Daily Life in COPD
Source: PLoS One. 2012 Jul 17;7(7):e41078. doi: 10.1371/journal.pone.0041078 (PMC3398871; doi:10.1371/journal.pone.0041078)
Supplement: Text S1 — Effects of Body Mass Index on Task-Related Oxygen Uptake and Dyspnea During Activities of Daily Life in COPD. (DOC) [file pone.0041078.s005.doc]

**Effects of Body Mass Index on Task-Related Oxygen Uptake and Dyspnea During Activities of Daily Life in COPD**

**Authors:**

Anouk W. Vaes^1,2^, Frits M.E. Franssen^1^, Kenneth Meijer^3^, Martijn W. J. Cuijpers^1^,

Emiel F.M. Wouters^1,4^, Erica P.A. Rutten^1^, Martijn A. Spruit^1^

**Affiliations:**

^1^Program Development Centre, CIRO+, Horn, the Netherlands;

^2^Physiotherapy, CIRO+, Horn, the Netherlands;

^3^Department of Human Movement Science, School for Nutrition, Toxicology and Metabolism of MUMC+, Maastricht, the Netherlands;

^4^Respiratory Medicine, MUMC+, Maastricht, the Netherlands.

**Corresponding author**

Anouk W. Vaes, MSc, PT

e-mail: anoukvaes@ciro-horn.nl

**METHODS**

The COPD patients who volunteered to participate in the present study were clinically stable (no exacerbation in past 4 weeks) and were on various (non-) pulmonary drug therapies (Table S1).

RESULTS

**Domestic ADLs**

Patients with COPD *versus* healthy elderly subjects

*Duration of ADL-protocol*

All subjects were able to complete ADL1 to ADL4 without stops during the performance of each ADL. COPD patients needed significantly more time to complete ADLs 1, 3, and 4 compared to healthy subjects (79±25 *vs*. 63±13s; 72±19 *vs*. 63±16s; and 104±31 *vs*. 72±20s; all p<0.001). Time to complete ADL2 was comparable: 115±21 *vs*. 125±28s.

Thirty COPD patients needed 20 to 145 s rest before they were able to start ADL5, and seven of them could not complete ADL5 due to exertional dyspnea.

*Task-related oxygen uptake*

Task-related oxygen uptake (mL/min) at the end of the 5 ADLs was similar between COPD patients and healthy subjects (Figure 1a). Consequently, patients performed the domestic ADLs at a significantly higher proportion of their peak aerobic capacity compared to the healthy subjects (Figure 1b). Moreover, COPD patients had a significantly higher task-related oxygen uptake per kilogram FFM compared to the healthy subjects (Figure S1a).

*Task-related ventilation*

Patients with COPD performed the domestic ADLs at a higher absolute ventilation (mean difference (95%CI) with healthy subjects for ADL1: 4.5 (1.7-7.2)L; ADL2: 6.0 (3.2-8.8)L; ADL3: 6.2 (3.3-9.1)L; ADL4: 7.5 (4.9-10.1)L; and ADL5: 8.2 (4.4-12.0)L; all p<0.001; Figure S1b). Moreover, patients had a signifiantly higher relative task-related ventilation compared to healthy elderly controls (mean difference (95%CI) for ADL1: 28.4 (21.4-35.4)%; ADL2: 31.6 (24.1-39.1)%; ADL3: 33.5 (25.5-41.4)%; ADL4: 33.5 (26.4-40.6)%; and ADL5: 39.7 (30.0-49.4)%; all p<0.001; Figure S1c).

*Borg symptom scores*

Patients with COPD reported significantly higher Borg symptom scores for dyspnea and fatigue and the end of each ADL compared to the healthy controls (Figure S1d and S1e).

COPD patients stratified by body mass index

*Duration of ADL-protocol*

All patients were able to complete ADL1 to ADL4 without stops, with no significant differences in time to complete the domestic tasks between BMI groups (Figure S2).

Thirty patients with COPD needed 20 to 145 s rest before they were able to start ADL5, while seven patients were not able to complete ADL5 due to exertional dyspnea. No differences were found between BMI groups.

BMI-related differences in data previous study

Analysis on data of our previous study showed that absolute oxygen uptake and absolute ventilation after the performance of domestic ADLs is significantly lower in underweight COPD patients compared to overweight or obese COPD patients (Figure S3a and S3b). Relative task-related oxygen uptake and ventilation were comparable between BMI groups (Figure S3c and S3d).

REFERENCES

1. Vaes AW, Wouters EF, Franssen FM, Uszko-Lencer NH, Stakenborg KH, et al. (2011) Task-Related Oxygen Uptake During Domestic Activities of Daily Life in Patients With COPD and Healthy Elderly Subjects. Chest 140: 970-979.

**FIGURE LEGENDS**

**Figure S1 Results of patients with COPD and healthy elderly subjects after performance of 5 simple domestic activities of daily life**

1. Task-related oxygen uptake per kilogram FFM (mL/min/kgFFM)
2. Absolute task-related ventilation (L/min)
3. Relative task-related ventilation (%peakVE)
4. Borg dyspnea scores (points)
5. Borg fatigue scores (points)

*p<0.001 COPD versus healthy elderly subjects

**Figure S2 Time to accomplish ADLs**

**Figure S3 Results after performance of simple domestic activities of daily life in patients with COPD after stratification for BMI**

1. Absolute task-related oxygen uptake (mL/min)

BMI>30 kg/m^2^ was significantly higher compared to BMI<21 kg/m^2^ at rest and after all ADLs (p<0.01) and compared to BMI 21-25 kg/m^2^ for ADL 1 and 2 (p<0.05). BMI 25-30 kg/m^2^ was significantly higher compared to BMI<21 kg/m^2^ at rest and after all ADLs (p<0.01). BMI 21-25 kg/m^2^ was significantly higher compared to BMI<21 kg/m^2^ at rest and after ADLs 2 and 5 (p<0.05).

1. Relative task-related oxygen uptake (%peakVO2)
2. Absolute task-related ventilation (L/min)

BMI>30 kg/m^2^ was significantly higher compared to BMI<21 kg/m^2^ at rest and after all ADLs (p<0.05).

1. Relative task-related ventilation (%peakVE)
